# Pest Management and Ochratoxin A Contamination in Grapes: A Review

**DOI:** 10.3390/toxins12050303

**Published:** 2020-05-07

**Authors:** Letizia Mondani, Roberta Palumbo, Dimitrios Tsitsigiannis, Dionysios Perdikis, Emanuele Mazzoni, Paola Battilani

**Affiliations:** 1 Department of Sustainable Crop Production (DI.PRO.VE.S.), Faculty of Agriculture, Food and Environmental Sciences, Università Cattolica del Sacro Cuore, Via Emilia Parmense 84, 29100 Piacenza, Italy; letizia.mondani@unicatt.it (L.M.); roberta.palumbo@unicatt.it (R.P.); 2 School of Plant Sciences, Department of Crop Science, Laboratory of Plant Pathology, Agricultural University of Athens, Iera Odos 75, 11855 Athens, Greece; dimtsi@aua.gr; 3 School of Plant Sciences, Department of Crop Science, Laboratory of Agricultural Zoology and Entomology, Agricultural University of Athens, Iera Odos 75, 11855 Athens, Greece; dperdikis@aua.gr

**Keywords:** *Lobesia botrana*, forecasting models, mycotoxins, insects, black aspergilli, *Aspergillus carbonarius*’ *OTA*

## Abstract

Ochratoxin A (OTA) is the most toxic member of ochratoxins, a group of toxic secondary metabolites produced by fungi. The most relevant species involved in OTA production in grapes is *Aspergillus carbonarius*. Berry infection by *A. carbonarius* is enhanced by damage to the skin caused by abiotic and biotic factors. Insect pests play a major role in European vineyards, and Lepidopteran species such as the European grapevine moth *Lobesia botrana* are undoubtedly crucial. New scenarios are also emerging due to the introduction and spread of allochthonous pests as well as climate change. Such pests may be involved in the dissemination of OTA producing fungi even if confirmation is still lacking and further studies are needed. An OTA predicting model is available, but it should be integrated with models aimed at forecasting *L. botrana* phenology and demography in order to improve model reliability.

## 1. Introduction

Ochratoxins are a group of toxic secondary metabolites produced by fungi. Ochratoxin A (OTA) is the most toxic member of the ochratoxin group as well as the most studied, and it has been classified as a group 2B carcinogen (i.e., possible human carcinogen) by the International Agency for Research on Cancer (IARC) [1]. OTA occurs at global level in a wide variety of agricultural products (e.g., cereals, grapes, coffee, cocoa, spices) as well as derived foods and beverages, including grape juice, wine and dried vine fruits (i.e., currants, raisins and sultanas) [2,3,4]. Wine was first reported to be contaminated with OTA in 1996 [5], and at present it is regarded as the second major source of human exposure to OTA, after cereals. OTA has been demonstrated to be nephrotoxic and associated with a fatal human kidney disease, Balkan Endemic Nephropathy (BEN) [6,7]. Acute toxicity of OTA is mostly due to nephrotoxicity and hepatotoxicity; it has also shown carcinogenic, immunotoxic, genotoxic, teratogenic and possibly neurotoxic properties [8]. Due to the risks for consumers associated with dietary exposure to OTA, maximum permitted levels (MLs) have been established for this mycotoxin in the EU in different grape products (i.e., 2 µg/kg in wine and grape juice; 10 µg/kg in dried vine fruits (currants, raisins and sultanas)) [9].

OTA is produced by several fungal species of the genera *Aspergillus* and *Penicillium*. These genera are part of the complex microflora of grapes together with other filamentous fungi (i.e., *Alternaria*, *Acremonium*, *Botrytis, Cladosporium* and *Rhizopus*) [10,11,12,13,14,15]. However, the main species responsible for OTA contamination of grapes belong to the genera *Aspergillus* section *Nigri*, the so-called black aspergilli [3,16].

Black aspergilli are often present in the soil and on grape berries during the entire crop production cycle, although they have difficulty penetrating healthy berries at early grape growth stages; their entry into the fruit is enhanced by skin damage caused by insect pests or other factors that may cause splitting of the berries such as rainfall or fungal infections (i.e., powdery mildew) [17]. Among these factors, insect pests are confirmed to play a major role in the fungal invasion of berries and their OTA contamination. However, despite the large number of insect species infesting grapes [18], only a limited number of them, e.g., the European grapevine moth *Lobesia botrana* (Denis and Schiffermüller) (Lepidoptera: Tortricidae) or mealybugs have been associated with OTA contamination [19,20].

The use of effective pest management strategies against grape moths has minimized the risk of OTA contamination in the grape chain [21,22,23]. However, global warming is likely to bring more frequent extreme climatic events that may lower the efficacy of OTA contamination mitigation measures due to their variable impact on fungi, insect pests, grapes and on their interactions. In addition, due to globalization new insect pests are emerging in vineyards worldwide and assessment of their potential involevement in OTA contamination has to be prioritized. In this context, the use of predictive systems for OTA risk has been recognized as a valid tool to support the optimization of grapevine planning and management practices along the value chain [24]. However, model validation requires a consistent set of quantitative data, whereas efforts are required to predict the impact of *L. botrana* and to integrate it in the modelling of OTA contamination and in mitigation measures.

Thus, the aim of this paper is to (i) revise the recently increasing available information in peer-reviewed literature on black aspergilli and OTA contamination in grapes and main insect pests that cause damage to grape berries and show their possible relation with OTA contamination (ii) to collect data on the phenology of *L. botrana* as well as ecological factors influencing its behavior with a view to modelling implementation on its influence on OTA contamination in grapes. The need for future research will also be highlighted.

## 2. Black Aspergilli and Ochratoxin A Production in Grapes

**Figure 1:**
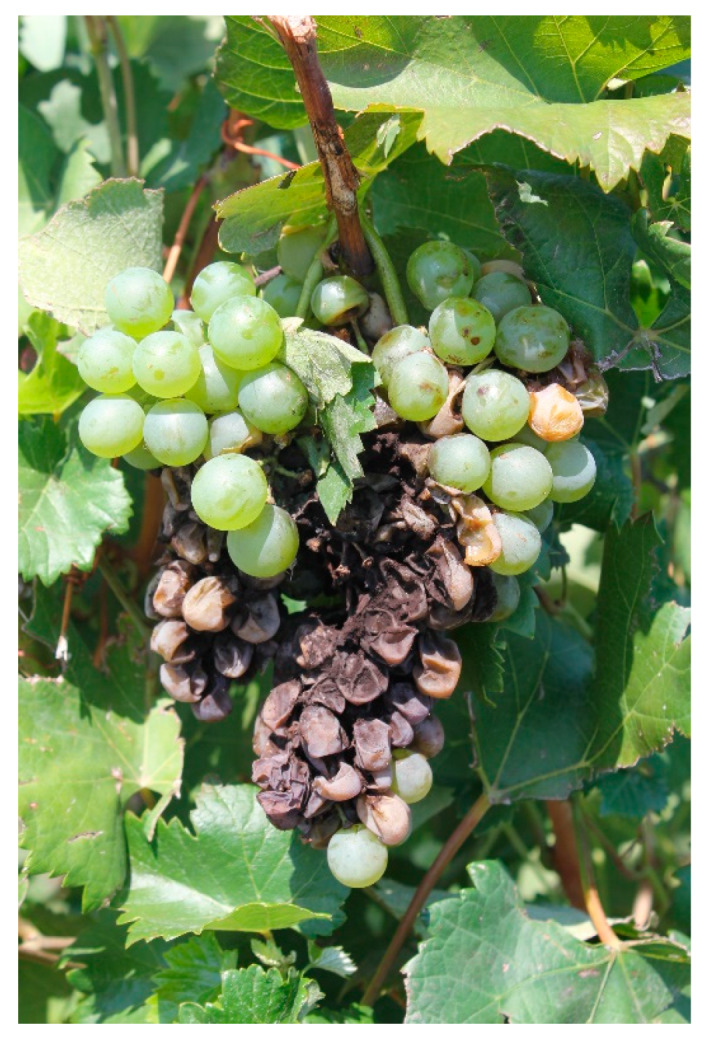
Grape bunch (cv Malagouzia) with Aspergillus rot caused by *Aspergillus* section *Nigri* (August 2018, Spata-Attiica-Greece).

The genus *Aspergillus* currently includes 185 species and is one of the most important genera in food spoilage and contamination [25]. More than 30 species of *Aspergillus* have been isolated from grapes in vineyards around the world, and *A. carbonarius* and *A. niger* aggregates, belonging to *A.* section *Nigri* are the most frequently reported species, accounting for 50–98.5% of all *Aspergillus* strains [26,27,28,29,30,31,32] as well as the most relevant species involved in OTA production and accumulation in wine [33,34,35] (Figure 1). 

### 2.1. Physiology and Ochratoxin A Production by Black Aspergilli

Black *Asperigilli* grow at temperatures between 8 °C and 45 °C. *A. carbonarius* is more sensitive to low and high temperatures with respect to *A. niger*. The optimum temperature for both species is around 30 °C with water activity (a_w_) = 0.96 and 0.98, respectively [36,37]; nevertheless, *A. niger* is confirmed as more thermophilic than *A. carbonarius* [29,38]. *A. niger* aggregate is more abundant and more frequently isolated from grapes than *A. carbonarius*, although only 2–20% of *A. niger* isolates are OTA producers [21]; on the contrary, nearly 100% of the isolates of *A. carbonarius* are toxigenic [39,40,41]. *A. carbonarius*, on the other hand, is very tolerant to high CO_2_ concentrations. Pateraki et al. (2007) demonstrated that a modified atmosphere composed by 25% of CO_2_ and 1% of O_2_ stimulates *A. carbonarius* growth whereas an atmosphere composed by 50% of CO_2_ and 1% of O_2_ slightly reduces growth capacity on grape juice medium at 0.85–0.93 a_w_ [42]. The optimal range of temperatures and a_w_ for OTA production in most isolates is 15 °C and 0.95-0.97 a_w_ or 20 °C and 0.98–0.99 a_w_, but also optimal temperatures ranging from 30 °C to 35 °C are reported for some isolates [37,43,44,45,46,47].

In addition, pH can influence the production of OTA in grapes and pH values ranging from 2 to 10 have been reported to be conductive for OTA production on CYA and YES media [48,49].

### 2.2. Ecology in Vineyard

Black aspergilli are present on berries from fruit set to ripening and their incidence increases towards harvest. Normally, they overwinter in soil and vine residues [2,50]. *A. carbonarius* is a saprophyte, so penetration of grape berries preferentially occurs after damage caused by insects, heavy rainfall and storms or other fungal infections (i.e., powdery mildew). The highest incidence of black aspergilli is recorded at fruit maturity, when high levels of sugar accumulate in berries, but infections can also start at berry setting [50]. Field trials have confirmed that the last 20 days of ripening are crucial for OTA synthesis in grapes [50].

Weather and region of origin can greatly influence OTA contamination [21,51,52]. Battilani et al. [16] found that the incidence of black aspergilli at harvest was related to the latitude and longitude of vineyards, and a positive gradient was detected moving from west to east and from north to south in Europe. Further, the incidence of infected berries was highest in the hottest and driest years.

### 2.3. Black Aspergilli and Ochratoxin A Production in Dried Grapes

“Dried grapes” commercially means dried vine fruits and they differ in name depending on the type of grape used: raisins and sultans from white seedless grapes, and currants from red seedless grapes. The average OTA contamination in dried grapes is usually much higher than in wine with values ranging up to 50–70 µg/kg [53]. During the drying process, the ratio between *A. carbonarius* and *A. niger* changes in favor of *A. carbonarius* [54], which is known to be the most efficient OTA producer, and is still active until a_w_ becomes unsuitable (a_w_ < 0.85) [55]. Most reports regarding OTA in dried grapes concern the Mediterranean area and the incidence of contamination can be up to 88% of the samples [54,56,57,58,59], greatly influenced by the geographic area and the year of production [60]. OTA contamination can be mitigated by SO_2_ treatments and a rapid drying process with temperatures higher than 30 °C. Moreover, the elimination of discolored berries and the frequent turning over of the fruits can greatly reduce the accumulation of OTA during the drying process [57].

### 2.4. Ochratoxin A in Grape Juice and Wine

The occurrence of OTA in grape juice is of particular interest for public health, because of the widespread consumption of this product by children who are its major consumers [61]. Despite this, few studies have focused on this topic. Zimmerli and Dick [5] detected OTA contamination in red grape juice in the Swiss market with a median concentration of 235 pg/mL. In Europe, in some cases OTA contamination levels in red grape juice are comparable to wine (5.26–7 µg/L) [53,62,63,64]. On the other hand, only 17% of white grape juice samples showed OTA contamination [65]. OTA has also been detected in a must derived product, “pekmez”, a condensed juice produced in Turkey, and 48% of the samples analyzed in the study exceeded the EU limit for safe consumption (2 µg/kg maximum level for grape juice) [66]. OTA accumulates in grapes during the cropping season, and it is commonly dectected after early veràison. White wine is definitely less prone to OTA contamination; the period between berry breakage and juice separation is very limited and the release of OTA from berry skin is therefore avoided. The red wine making process does not lead to an increase in OTA compared to berry contamination and some reduction is reported, despite its release from grape skin during maceration. Clarification, alcoholic and malolactic fermentation can contribute to OTA reduction. Impact on wine color is the principal factor for compounds used for clarification; those applied in white wine greatly influence wine color and OTA adsorption too, but their impact is unwanted in red wine, where the role of the color is crucial. Furthermore, a significant impact of yeasts and lactic bacteria on OTA content, during alcoholic and malo-lactic fermentations, was observed only when selected strains were added, but this is increased if grapes are contaminated. Combined control strategies and timely production of juice and wine can result in lower OTA accumulation [21].

## 3. Main Insect Pests on Grapes

Several insect species can be considered grape pests and their importance is usually variable in different environments [67]. The pest status and the corresponding impact of insects feeding on grapes depends greatly on their feeding habits. Indeed, two main approaches can be recognized linked to a different type of mouthpart structure: sucking or chewing. In the first case, phytophagous species use modified mouthparts to reach the vascular system or simply to empty cells in the parenchyma or in the mesophyll. In several cases the true economic damage caused by these pests is not due to sap or cytoplasm removal; in fact, honeydew production or pathogen transmission (viruses or phytoplasmas) are much more important. The chewing species remove plant tissues, usually creating more or less extended injuries in plant tissues. In this case, grape tissue removal can also be of limited importance if compared to the possibility that wounds can open a penetration route for many different pathogens producing berry rot.

In the history of grape cultivation, several significant examples of changes in phytosanitary problems have been reported and often business practices have greatly modified the importance of a specific pest. For example the grape phylloxera (*Daktulosphaira vitifoliae* (Fitch), Hemiptera: Phylloxeridae) upon its arrival in Europe, approximately in the middle of the nineteenth century, impacted significantly on grape cultivation [67,68], making grafting mandatory. More recently, the introduction of *Scaphoideus titanus* Ball (Hemiptera: Cicadellidae), likely in mid-twentieth century France [69], forced the modification of pest management strategies in the areas where this leafhopper is distributed [70]. Some other species, usually not considered of phytosanitary importance, are changing their pest status. The most recent examples are the honeydew moth *Cryptoblabes gnidiella* (Millière) [71] and mealybugs that are having a greater and wider impact [72,73,74]. However, the role of temperature increase, as the result of new climate scenarios, in changing the relevance of different pests, is still under debate [75,76]. Experimental approaches have pointed out contrasting effects on different life traits of a pest such as *L. botrana;* increasing temperatures have reduced the length of the larval stage and increased both survival rates and the ability of larvae to move fast to avoid natural enemies. On the other hand, energy reserves (lipid accumulation) and the activity of some enzymatic systems like phenoloxydases involved in immunity response towards parasitoid eggs have been reduced [77].

Such events have modified, and are still modifying, the importance of traditional pests like vine moths. In European vineyards, undoubtedly the major role as key pests is still played by twoLepidopteran species: the European grapevine moth *L. botrana* and the grape berry moth *Eupoecilia ambiguella* (Hübner) (Lepidoptera: Tortricidae).

Both moths are polyphagous pests that attack many botanical species with berry and berry-like fruits [78]. Both species have a comparable behavior and they attack grapes producing very similar damage, but they do not completely share an ecological niche; *L. botrana* is more thermophilic and *E. ambiguella* prefers a fresh and cold climate [79]. The latter was the most important pest in European vineyards until the first decades of the 20th century, but in many areas it is being replaced by the former species that has expanded its prevalence area from the Mediterranean Basin to Switzerland, Austria and southern Germany [80]. Nowadays *L. botrana* distribution covers a geographical area that includes Central Europe, the Mediterranean Basin, southern Russia, Japan, the Middle East, and northern and western Africa [81]. *L. botrana* is still enlarging its distribution area and there were reports of new area sof colonization inChile in 2008 and Argentina in 2010. It was also reported in California in 2009 [82], but in this area, after a huge phytosanitary effort, it is now considered eradicated [83]. The distribution range of *E. ambiguella* is from Britain to Japan, from the Mediterranean Basin to the Scandinavian countries; therefore, much wider than the grapevine-growing regions [84].

### 3.1. Life Cycle and Impact of Grape Moths

*L. botrana* share with *E. ambiguella* a similar life cycle and behavior. Both are multivoltine species with facultative diapause even if the latter usually completes a smaller number of generations. On *Vitis vinifera, L. botrana* develops 2–4 generations per year depending on latitude and climate [85,86]. According to different literature sources, the temperature range for its development is between 8 °C and 34 °C, with an optimum in the range of 28–30 °C. In most cases, the effect of temperature on its developmental rate has been evaluated in lab conditions, feeding the insect with an artificial diet [87,88]. It is frequent to observe a tendency to increase the number of generations per year in many European and Mediterranean areas [89,90,91], but an increasing number of generations does not mean necessarily an increased impact of the pest on grape. Indeed, the increasing number of generations could have a significant fitness cost for the pest. In fact, if the last generation larvae do not complete their development before harvest, they can be irreversibly removed from the population pool [85,89,92]. *E. ambiguella* usually has two generations per year, even if a third generation has often been detected in warmer areas or years [93,94].

A common trait to both species is the female habit of laying single eggs instead of clusters. This is considered an evolutionary advantage increasing the chance for eggs to avoid natural enemies [18]; however, it makes visual monitoring in field more difficult.

**Figure 2:**
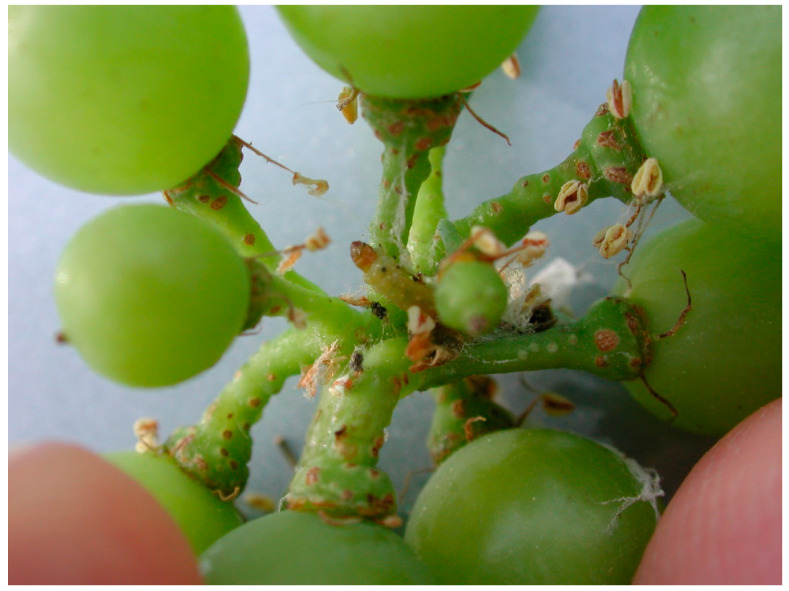
First generation *L. botrana* larva and nest among developing berries (June 2018, Riccagioia, Oltrepo pavese-Italy).

In both species, the females, developed from the overwintering pupae, after mating, lay from 50 to 80 eggs on flower buds [78]. Then, the first-generation larvae are anthophagous and feed on pre-bloom flowers. During their development, these larvae use silk to build up a “nest” or “glomerulus” which is used as a refuge against adverse conditions (unfavourable temperature, predators and parasitoids). First generation attacks are not economically important for most grape cultivars [95,96] (Figure 2). In the following generations, eggs are laid singly on berries and new born larvae and after a short wandering period, penetrate berries. With their feeding activity, larvae produce “direct damage” which varies in severity depending on grape cultivar, ripening stage and larval age; further, a single larva usually damages several berries [97]. Depending on the water content and grape ripening stage, larvae can erode berries superficially or dig deeper inside. The damage level can be linked also with the oviposition preferences of gravid females. Waxy layers present on berries can act as specific attractants and this can account, at least partially, for cultivar susceptibility [98], but olfactory as well as visual stimuli can also play an important role [99,100].

“Direct” damage is a prerequisite for “indirect” damage, which is linked to the interaction of moth larvae with some fungi and it is often considered the most important from an economic point of view. Indeed the link between the feeding activity of larvae and resulting fungal damage has been demonstrated for *L. botrana,* but less/no data are available in the literature for *E. ambiguella* or any other moth species whose larvae can attack grape berries [101].

One of the best known examples regards grey mold (*Botrytis cinerea*). The fungus can use wounds produced by larvae to enter grape berries, but larvae are also involved in conidia delivery [102]; in addition, grey mold conidia can survive in the gut of the larvae and can be recovered viable from the faeces [103].

It has also been demonstrated that direct damage to the berries produced by moth larvae can greatly favor sour rot mediated by secondary pests such as *Drosophila* sp. [104].

### 3.2. Lobesia botrana and OTA

The link between *L. botrana* larval activity and OTA-producing *Aspergillus* spp. was pointed out in Apulia some years ago [19]. Authors reported significant differences in the number of colony forming units per g of berries (CFU/g) as well as in the OTA content among healthy berries, berries with *Aspergillus* rot symptoms and berries with *L. botrana* larvae damage and *Aspergillus* rot. Further, from asymptomatic berries collected from bunches with *Aspergillus* symptoms or with *L. botrana* damage, CFU counted were roughly 12 times higher in berries from bunches attacked by *L. botrana*. High variability in OTA content at harvest was detected, but the highest concentration (up to 681 ng/g) was always measured in berries from bunches with *L. botrana* attack symptoms. Further field trials pointed out and confirmed the association between *L. botrana* activity and the increase of fungi incidence and consequent OTA contamination in different environmental and agronomic situations [105,106]. In fact, high relative humidity, rainfall and temperature at the ripening period are very favorable for both *L. botrana* infestations and for OTA production indicating the key role that *L. botrana* has in the OTA contamination levels in grapes [105].

Tsolakis and collagues [107] evaluated the link between *L. botrana* and OTA contamination in Sicily in an organic vineyard and confirmed the OTA content was much higher (about 500 times) in bunches with *L. botrana* attacks in comparison with healthy ones. Nevertheless, in years with adverse climatic conditions for the European grapevine moth (high temperatures and low relative humidity), the ratio was only four times and the OTA concentration dropped from 20 µg/kg to 0.055 µg/kg.

### 3.3. Other Insect Risk

Currently, in the Mediterranean area, only *L. botrana* is reported to be a pest linked to OTA accumulation in grapes [19]. Indeed, no specific demonstration of the involvement of *E. ambiguella* or other moths like *C. gnidiella* in OTA contamination exist till now, but considering that such species have a similar feeding behavior, it is very likely that these moths could play a comparable role in enhancing OTA contamination. Nevertheless, considering that any insect causing wounds on grapes by feeding or ovipositing can be potentially linked to OTA, the list of species may be much longer. Experimental evidence exists for the fly *Anastrepha fraterculus* (Wiedemann) (Diptera: Tephritidae) in Brazil. As many phytopathogenic fungi have been found in all the body parts of the adults of this insect, it has been suggested that the fly can serve as a mechanical vector of spores [108]. A significant increase of bunches infected by *B. cinerea*, *Glomerella cingulata*, and microorganisms of sour rot was demonstrated after oviposition wounds by *A. fraterculus,* even if no specific OTA assessment was performed [109]. Similar observations have also been carried out on *Polistes dominulus* (Christ) (Hymenoptera: Vespidae) in lab conditions, demonstrating the attitude of this species to facilitate sour rot diseases increasing host susceptibility and transmitting microbial communities to grapes, including *Aspergillus* spp. and *Botrytis* spp. [110].

Recently, mealybugs like *Planococcus ficus* Signoret (Hemiptera: Pseudococcidae) have been reported to play a role in OTA contamination as demonstrated by Chiotta et al. in Argentina working on red wine cultivars (Malbec, Merlot and Cabernet Sauvignon) [20]. According to their findings, *A.* section *Nigri* incidence and OTA concentration were higher in damaged berries than in healthy ones regardless of the cultivar and the growing period. The authors also suggest a significant involvement of ants in distributing black aspergilli as they feed on *P. ficus* honeydew that may also be a good substrate for fungal growth (Table 1).

New potential factors favouring OTA production in grapes may be the invasive insect pests that feed on berries and recently established in the Mediterranean area as well as in other regions worldwide [111]. The spotted-wing drosophila, *Drosophila suzukii* Matsumura (Diptera: Drosophilidae) inserts its eggs in berries by punctures made by its ovipositor exposing fruits to secondary pathogens. Larvae feed under the skin creating tunnels in the fruit flesh that results in collapse and rotting [112,113]. Attacks by this pest have been associated with fungi infections [113,114,115]. The brown marmorated stink bug, *Halyomorpha halys* (Stål) (Hemiptera: Pentatomidae), feeds by piercing the berries and infests vineyards mainly during harvest [116,117]. Its damage may be associated with disease infections of fruits including grapes [118,119]. Another pest that may play a role in the OTA contamination of grapes is the Mediterranean fruit fly, *Ceratitis capitata* (Wiedemann), which has been reported to cause significant damage and outbreaks in table grapes of the Mediterrannean area, South Africa and Brazil [120,121,122,123]. These pests may be involved in OTA producing fungi dispersal, therefore their role should be taken into account in pest control strategies to be incorporated in OTA mitigation measures [124].

## 4. Control Measures for Black Aspergilli and *Lobesia botrana*

### 4.1. Control of Black Aspergilli

Agricultural practices are reported as the main tools to control OTA contamination in grapes [126] and good agricultural practices (GAPs) can reduce up to 80% OTA presence in wine [61,127] (Table 2).

Hocking et al. [128] observed that minimizing *Aspergillus* spp. inoculum in soil, via irrigation and pruning by maintaining constant soil moisture and reducing dead berry falling, respectively, can greatly reduce infections and OTA contamination during the cropping season. The trellising system used may impact the rate of bunch infection. Clusters closer to the soil seem to be more frequently infected, but the effect of proximity to the soil on OTA content has not yet been confirmed [21,129]. Furthermore, a correct management of canopy, pruning and irrigation—so that bunches are not exposed to sunburn, have a more open structure and berries are less susceptible to splitting—can lead to lower *Aspergillus* spp. infection and OTA accumulation. 

Correct vineyard management strategies for reducing berry damage, by controlling via chemical and biological treatments fungi colonization and insect damage, can lead to a significant decrease in OTA accumulation in grapes [19,61,130].

Both biological and chemical treatments aimed at controlling black aspergilli are important in the achievement of OTA reduction in grape production. Chemical treatments are effective in reducing fungal growth and consequently OTA content in berries. Cyprodinil in combination with fludioxonil was confirmed as the most effective fungicide in all trials conducted in France, Spain, Italy and Greece [131,132,133,134]. Other active ingredients showed the capacity to reduce fungal growth in grape bunches in field trials such as pyrimethanil, fluazinam, iprodione and mepanipyrim [106].

Biocontrol Agents (BCAs) are increasingly attracting interest as a sustainable means of disease control. In recent years, it has been demonstrated that certain microorganisms can effectively reduce Aspergilli growth in pre-harvest. De Felice et al. [135] and Dimakopoulou et al. [136] tested the efficacy in vitro and in vineyards of naturally occurring *Aureobasidium pullulans* strains against Aspergilli. Bleve et al. [137] also demonstrated the efficacy of *Cryptococcus laurentii* in controlling OTA producing fungi. BCAs can also have a role in reducing post-harvest diseases and OTA contamination *Kluyveromyces* and *Saccharomyces* yeast were evaluated by Ponsone et al. [138] and Nally et al. [139] both in vitro and in situ preventing ochratoxigenic fungi. Droby et al. [140] stated that applying a combination of BCAs with different mechanisms of action and under favourable conditions for their colonization and growth can lead to excellent results against a large spectrum of fungi. Finally, all pre- and post-harvest biocontrol strategies were recently revised by several authors and they confirmed their high potential in OTA prevention [141,142,143].

Time of harvest is another important factor in determining OTA content in wine. In fact, it is well known that *Aspergillus* contamination is quite low in immature grapes, due to the hostile environment for spore germination [144], but from veraison to harvest, sugar content increases and berry skin becomes softer, so berries gradually become more susceptible to the attack of fungi and more suitable for OTA production. For this reason, delaying harvest can lead to a higher risk of OTA contamination [50,128].

### 4.2. Control of Lobesia botrana

Due to the importance of *L. botrana* in enhancing OTA contamination in grapes, the application of phytosanitary measures against the pest can be a promising approach as highlighted also in the system “corn-European Corn Borer-*Fusarium*” [146]. The management of larvae in open field can reduce OTA concentrations in grapes in integrated as well as in organic farming [105,106,125].

The impact of pest control varies significantly between years and growing areas. In fact, interaction with climatic conditions is considerable and can impact on the pest population; also, the effect of insecticide applications can be completely hidden [105,125]. As an example, dry and hot summers can have extremely negative effects on eggs that do not survive temperatures above 34.5 °C; therefore, pest control in dry and hot summers may be not be necessary due to the natural low level of the pest population [78]. Nevertheless, In fact, interaction with climatic conditions is considerable and can impact on the pest population; also, the effect of insecticide applications can be completely hidden [105,125]. monitoring should be continous to avoid damages close to harvest and this must be seriously considered.

**Figure 3:**
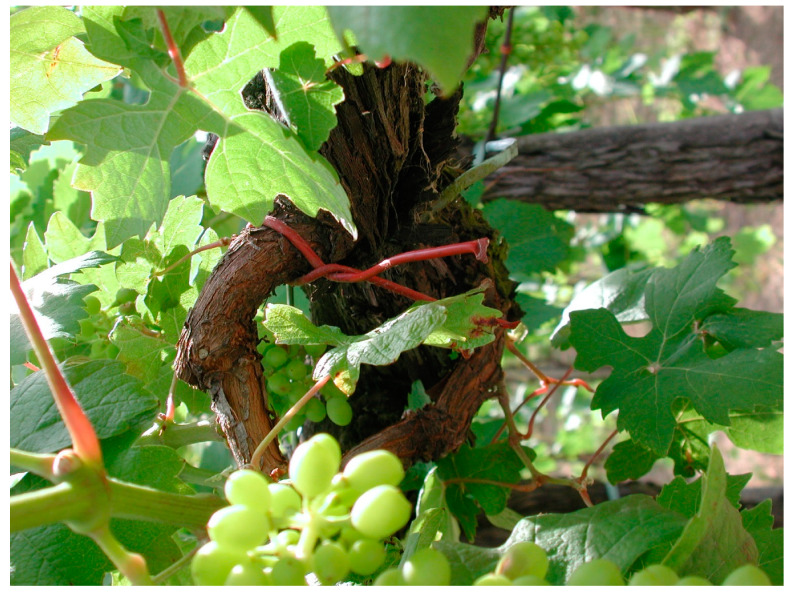
Mating disruption dispenser (June 2018, Riccagioia, Oltrepo pavese–Italy).

Nowadays, phytosanitary measures against *L. botrana* still greatly rely on chemical insecticide applications, but alternative, low impact approaches are also available [124,147,148,149] (Figure 3). Learning from natural events, mating disruption (MD) is pursued; sex pheromones used by virgin females to attract selectively conspecific males can be delivered in the field in amounts largely exceeding their natural occurrence (several hundred mg/day) preventing males from detecting natural pheromone plumes emitted by virgin females (a few ng/day). In this way, matings are greatly reduced and population declines. Indeed, in many areas, classic MD, based on several hundred plastic or biodegradable pheromones dispensers/ha, or modern MD, based on just a few automatic dispensing devices/ha, are widely used and their application rates are constantly increasing [147,150,151,152]. MD enables “conservation biological control”, whereby parasitoids and other natural enemies are allowed to flourish and contribute both to control target and non-target pests. Outbreaks of secondary pests sometimes observed with MD application are related by some authors to limited insecticide use; this needs further evaluation but so far this is not limiting MD success or efficacy [151]. However, outbreaks of mealybugs or *Drosophila suzukii* may occur in vineyards in which MD is applied [132] and thus, monitoring of their populations is required to prevent OTA contamination in grapes.A classic and well-known microbial control agent like *Bacillus thuringiensis* (BT) is an interesting tool against grape moth larvae [153]. Recent formulations have demonstrated an efficacy comparable with chemical insecticides. The counterpart is represented by the larval feeding behavior of *L. botrana*, that has a tendency to dig inside the berries thus avoiding BT toxin ingestion [154]. For this reason, an accurate evaluation of the timing of applications is crucial to precisely target first instar larvae as soon as they emerge from the eggs.

Biological control is considered an interesting tool even if it has not been fully exploited. Knowledge of the effect of some key factors (host plant, farming practices at the field scale, landscape context, as well as climate change), affecting moth-natural enemies relationship, would improve the efficiency levels of biological control strategies. This would help growers and stakeholders to significantly reduce chemical input in vineyards [18]. The possibility of biological control is also greatly influenced by the general temperature increase that produces contrasting effects on the moth–parasitoid system. Higher temperatures reduce the vulnerability window of *L. botrana* to larval parasitoids as the moth larvae grow faster thus increasing the chance to escape search by natural enemies, but on the other hand, such higher temperature can downregulate behavioral and immune traits that confer resistance to parasitoids [77,155].

## 5. Predictive Models

The need to improve management strategies and more recently demand for predicting the potential effect of climate change, has recommended the development of mathematical models to forecast OTA and pest development.

### 5.1. Black Aspergilli Predictive Models

Several in vitro studies regarding the impact of ecological conditions on black aspergilli have been used for modelling, but just aimed at describing single steps of the fungal infection cycle, mainly growth and toxin production [29,156,157,158,159,160,161]. The resulting empiric models are a useful base for further modelling steps, but not helpful for grape management. The only example of mechanistic model able to predict OTA risk in grapes was developed by Battilani et al. [24]. Using hourly data on air temperature and humidity and rainfall as input, the model delivers a risk assessment, a probability of grape contamination above the legal limit of 2 µg/kg during the growing season and at harvest. The model validation step is still missing because of the lack of a consistent data set of georeferenced grape contamination data with the related meteorological information. Once validated, this model will be suitable also to deliver risk predictions under climate change scenarios and could include other relevant factors like the interaction of black aspergilli with pest insects or the cropping system.

### 5.2. Lobesia botrana Predictive Models

Several approaches have been adopted to develop predictive models for *L. botrana*. The simplest model attempts to predict pest phenology, mainly adult moth flight periods, using degree-days [162,163,164,165,166]. This method is straightforward and quite useful, but usually the applicability of such models is strictly local [167]. More sophisticated, but still phenological and empirical models, simulate pest development using linear or non-linear functions [168]. A fully functional application of a model only using daily temperature as input and based on a theoretical approach, published several years ago [169], is currently used in the Emilia-Romagna region (Northern Italy). The model outputs are, on a daily basis, the cumulated percentages of oviposition, of hatched larvae, of newly developed chrysalis and of hatched adults as well as the percentages of eggs, larvae, chrysalis and adults relative to a given generation. The model is used to predict up to the third generation of the pest and it is considered by farmers and technicians a very useful tool, above all, to get a reliable prediction of the occurrence of different pest developmental stages in the field and to help in choosing the best timing for sampling and management applications [170,171,172].

Much more sophisticated models have been developed with the aim to predict the phenology, but also the demography, of the moth stages starting from the abundance of adults at the beginning of the flight period, using temperature as the main driving factor and including mortality estimation [173,174]. Linking physiologically-based demographic models (PBDMs) for grape development such models have also been evaluated to simulate and estimate the expansion area of the pest [175].

The application of simulation models—evaluating not only the phenology of the pest, but also considering mortality factors or spatial distribution—could be quite useful as support to optimise moth pest control [85,175]. However, so far no practical and large scale application of such models exists.

## 6. Conclusions and Prospective

A model able to predict the risk of OTA contamination in grapes above the legal limit in force in Europe (OTA-grapes) is available and any effort to link its forecasting ability with models describing other aspects/parts of the system (grapes-pest-environment) will certainly improve grape cultivation sustainability and production safety. For example, *L. botrana* plays a key role in OTA contamination of grapes and, therefore, models that report on its phenology and particularly on its larval emergence, if combined with OTA-grapes, could improve model prediction performance and support more reliable decision making in the control of black aspergilli infections. Furthermore, the involvement in OTA contamination of the other rather common grape pest, *E. ambiguella*, should be examined and quantified, since this pest behaves in a very similar way to *L. botrana* in its interaction with the host plant, whereas its distribution is very wide. Modelling of *E. ambiguella* phenology and population dynamic is very limited, but it should be considered in the framework of changing climate that can deeply affect its prevalence in grape growing areas. In model implementation, temperature is a mandatory input as it is always the most significant driving force for insect development. On the other hand, variables such as rainfall and relative humidity can significantly affect pest mortality and should be included in more sophisticated models. Knowledge regarding the impact of increasing CO_2_ on pest phenology is instead largely unknown, but it should be studied because CO_2_ is significantly increasing as the climate changes. CO_2_ impacts on all the components of host–parasite systems affecting the host, the parasite and the beneficial organisms in different ways [176]. An holistic approach should be applied in the future, leading to a greater role for predictive models in accounting for interaction and combination of multiple factors of the vineyard system, including pest and disease control strategies.

The potential of newly emerged pests, to be involved in OTA-producing fungi dissemination, introduces new challenges for the prediction of OTA contamination and OTA control in grapes. Their role demands urgent investigation and clarification because our knowledge of the phenology or the impact of these pests on grapes is still limited. At the same time, pest management strategies need to be modulated to integrate the new threats in vineyard IPM, limiting eventual negative impacts on eco-friendly approaches and widely adopted strategies.

## Figures and Tables

**Table 1:** Current information on insect pests and ochratoxin A (OTA) contamination in grape.

Pest	Geographical Area	Cultivar	Field/Lab Study	OTA Level	Berry Damaging Instar	References
*Lobesia botrana*	Apulia (Italy)	Bombino NeroUva di Troia	Field	108 ng/g (2001) 5 ng/g (2002)	Larvae	[19]
*Lobesia botrana*	Sicily (Italy)	Malvasia di Candia	Field	20 µg/kg	Larvae	[107]
*Lobesia botrana*	Apulia (Italy)	PrimitivoNegramaro	Field	1.13––2.89 ng/mL (2005/untreated plots) 0.47–0.98 (2005/insecticide treated plots)	Larvae	[125]
*Anastrepha fraterculus*	Brazil	Italia	Lab study	(*)	Adults (oviposition) and larvae	[109]
*Polistes dominulus*	USA	Thompson Seedless	Lab study	(*)	Adults	[110]
*Planococcus ficus*	Argentina	Red wine cultivars	Field study	51 µg/kg (2007) 9 µg/kg (2009)	Adults and nymphs	[20]

(*) These insects pests are mentioned to be involved in favouring fungi dispersal but no OTA measurement has been reported.

**Table 2:** Current information on Good Agricultural Practices and other factors that affect the ochratoxin A (OTA) contamination in grape.

Crop Attributes/Agricultural Practice	Notes	References
Crop type/processing	Dried grapes > grape juice > red wine > white wine	[5,145]
Geographical area vineyards location	Southern and eastern European regions are more susceptible than northern and western regions	[3,51,52]
Trellising system- proximity of berries to the soil	In vineyards in the south of Europe, bunches were 40–60 cm above soil, whereas in vineyards in the north were 150 cm above the soil	[129]
Pruning	Bunches must have an open structure with berries not being exposed to sun burn, reduce dead berry falling	[128]
Irrigation	Irrigation should be scheduled to prevent berry splitting	[128]
Harvest time	Harvest date must be set as soon as grape is mature	[50,128]
IPM	OTA occurrence in wine can decrease up to 80% using appropriate fungi and pest management	[61,127]
